# Dynamical Oligomerisation of Histidine Rich Intrinsically Disordered Proteins Is Regulated through Zinc-Histidine Interactions

**DOI:** 10.3390/biom9050168

**Published:** 2019-04-30

**Authors:** Carolina Cragnell, Lasse Staby, Samuel Lenton, Birthe B. Kragelund, Marie Skepö

**Affiliations:** 1 Division of Theoretical Chemistry, Department of Chemistry, Lund University, P.O. Box 124, SE-221 00 Lund, Sweden; Carolina.Cragnell@teokem.lu.se (C.C.); samuel.lenton@teokem.lu.se (S.L.); 2 Structural Biology and NMR Laboratory, The Linderstrøm-Lang Centre for Protein Science and Integrative Structural Biology at University of Copenhagen (ISBUC), Department of Biology, University of Copenhagen, 2200 Copenhagen N, Denmark; lasse.staby@bio.ku.dk (L.S.); bbk@bio.ku.dk (B.B.K.)

**Keywords:** Intrinsically disordered proteins, oligomerisation, Histatin, NMR, SAXS, computer simulation

## Abstract

Intrinsically disordered proteins (IDPs) can form functional oligomers and in some cases, insoluble disease related aggregates. It is therefore vital to understand processes and mechanisms that control pathway distribution. Divalent cations including Zn^2+^ can initiate IDP oligomerisation through the interaction with histidine residues but the mechanisms of doing so are far from understood. Here we apply a multi-disciplinary approach using small angle X-ray scattering, nuclear magnetic resonance spectroscopy, calorimetry and computations to show that that saliva protein Histatin 5 forms highly dynamic oligomers in the presence of Zn^2+^. The process is critically dependent upon interaction between Zn^2+^ ions and distinct histidine rich binding motifs which allows for thermodynamic switching between states. We propose a molecular mechanism of oligomerisation, which may be generally applicable to other histidine rich IDPs. Finally, as Histatin 5 is an important saliva component, we suggest that Zn^2+^ induced oligomerisation may be crucial for maintaining saliva homeostasis.

## 1. Introduction

It has been firmly established that intrinsically disordered proteins (IDPs) regulate many biological processes [1]. IDPs do not rely on a well-defined three-dimensional structure to perform their cellular functions but instead sample a heterogeneous ensemble of conformations [2,3]. The functions of IDPs often involve interactions with other macromolecules [4]. These interactions can be regulated through biological processes such as post-translational modification [5,6] but also through interactions with other molecules including divalent metal ions such as Ni^2+^, Cu^2+^, and Zn^2+^ [7,8]. In many cases these interactions are directly related to the biological function of the protein [9]. For example, a group of intrinsically disordered phosphoproteins including casein and osteopontin interact with calcium phosphate in milk through phosphorylated residues, thereby stabilising the fluid [10,11,12,13]. Another example is statherin, a saliva protein that transports calcium and magnesium to the enamel, a crucial process required to maintain the mineral phase of teeth [14,15].

Under physiological conditions some IDP monomers can undergo oligomerisation driven either by self-association or through interaction with multivalent ions and biological macromolecules such as RNA or DNA [16]. The process of oligomerisation can be functionally important but may in some cases lead to the formation of insoluble disease-related aggregates [17,18]. An example of this is the histidine rich Aβ peptide where conformational changes of the protein can result in the formation of toxic soluble dimers, which in turn oligomerise, resulting in the formation of insoluble protein aggregates that are the hallmarks of Alzheimer’s disease [18,19]. In the case of Aβ, oligomerisation is driven by the interaction between the N-terminal Aβ 1-16 region and Zn^2^+ ions [20,21].

**Figure 1:**
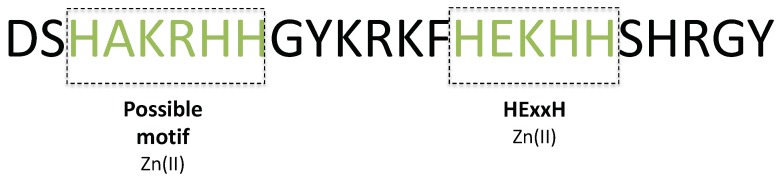
**Primary structure** of Hst5 with two of the zinc-binding motifs highlighted. The suggested possible zinc binding motif will be further discussed in the results section.

Histatin 5 (Hst5) is another histidine rich peptide found in saliva where it acts as first line of defence against oral candidiasis caused by Candida albicans [22,23,24]. In solution, Hst5 behaves as an IDP [25,26], but secondary structure elements can be induced under certain conditions [22,24,27]. Inherently, saliva contains various transition metals such as calcium, magnesium, zinc, nickel, copper, and iron [28]. Hst5 has been shown to bind Zn^2+^, Cu^2+^, and Fe^2+/3+^ ions [29,30,31], and it possesses defined metal binding motifs for copper (DSH, termed the ACTUN motif), nickel (DSH, ACTUN motif) [29], and zinc (a HExxH motif) [30]. Figure 1 displays the zinc-binding motif as well as the possible zinc-binding motif we suggest as a result of this article, to be discussed later.

Recent experimental work has shown that the interaction between Aβ and Zn^2^+ proceeds through two consecutive steps [20]: (i) primary Zn^2^+ binding by side chains of the histidine residues H6, E11, H13, and H14 for a single peptide, where the latter three originates from a zinc motif similar to the one present in Hst5, and (ii) dimerisation via zinc coordination of residues E11 and H14 in the zinc-binding motif. Hence, after the dimerisation seed is formed, further build-up of oligomers is due to Zn^2+^-histidine interactions through residues H6 and H13, see Figure 8 in the article by Polashakov et al. [20]. Further, Barnham et al. [32] showed that the Zn^2+^-induced oligomers of Aβ ranged in size from monomers to octamers, with one to three metal ions per peptide molecule [30], and with a higher affinity for Cu^2+^ compared to that of Zn^2+^.

In this paper, we widen the studies of the interactions between IDPs and divalent ions with an emphasis on Zn^2^+. We address the structural effects of Zn^2+^-binding to Hst5 with the overall aim to investigate if any general behaviour could be captured regarding metal ion induced oligomer formation of IDPs. For that purpose, parallels will be made to Aβ. By small angle X-ray scattering (SAXS) in combination with nuclear magnetic resonance (NMR) spectroscopy, isothermal titration calorimetry (ITC), and coarse-grained Monte Carlo (MC) simulations, we have confirmed that Hst5 binds Zn^2+^ and causes oligomer formation. By studying five different variants of Hst5, designed to elucidate the effect of dynamic histidine coordination, we suggest a possible mechanism for oligomer formation, and we hypothesise this to be a general mechanism for oligomerisation in the presence of divalent cations of histidine rich IDPs.

## 2. Materials And Methods

### 2.1. Sample Preparation

**Figure 2:**
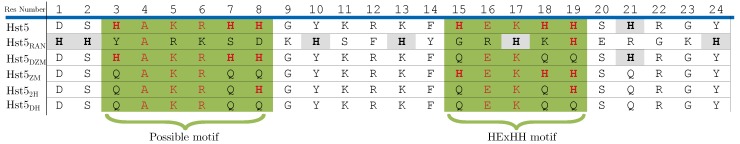
**Primary structures of Hst5 and variants**. The grey shaded areas denote the histidines and the yellow shaded area with red letters denote the known zinc-binding motif. Hst5_RAN_—random distribution of histidines, keeping the total number constant. Hst5_DZM_—The histidines in the zinc-motif have been replaced by glutamines. Hst5_ZM_—The histidines of the zinc-binding-like motif have been replaced by glutamines. Hst5_2H_—Two histidines in the zinc-motif have been replaced by glutamines, and only one of four histidines are left in the rest of the amino acid sequence. Hst5_DH_—All the histidines are replaced by glutamines.

Hst5 was purchased from American Peptide Company, CAS: [72-2-25] as well as Genemed Synthesis, Inc with a purity between 97.0–99.0%. The five variants were purchased from TAG Copenhagen A/S, Denmark, with a purity of 97.63%, 97.27%, 97.84%, 96.02%, and 96.15% for Hst5_RAN_, Hst5_DZM_, Hst5_ZM_, Hst5_2H_, and Hst5_DH_, respectively, measured by HPLC. For further explanation regarding denotation of the variants, please see Figure 2.

The 20 mM MES buffer (Sigma with a purity of >99.5Milli-Q water, and pH was adjusted with NaOH. The ionic strength of the solutions was adjusted with NaCl (Scharlau with a purity of >99.598.5 was filtered through a 0.2 μm hydrophilic polypropylene membrane (Pall Corporation, New York, NY, USA). After mixing the protein powder with the buffer, a concentration cell (Vivaspin 20, MWCO 2 kDa, Prod. No. VS2002, Sartorius Stedim Biotech GmbH, Goettingen, Germany), was used to remove low molecular weight impurities including ions from the freeze-dried sample. The samples were rinsed with buffer corresponding to at least 30 times the sample volume. To ensure exact match of the background, the samples were dialysed for 24 h (Slide-A-Lyzer MINI, MWCO 2 kDa, Prod. No. 69580, Thermo Scientific, Waltham, MA, USA).

### 2.2. SAXS

Prior to the SAXS measurements, the samples were centrifuged with an ultracentrifuge (rotor TLA55) at 13,000 rpm for at least 30 min to remove large aggregates. A Nanodrop spectrophotometer was used to measure the protein concentrations, ε = 2560 M^−1^cm^−1^, λ = 280 nm, immediately prior to SAXS measurements. To obtain the form factors at all ionic strengths, and decent signal to noise ratios, the protein concentrations were set to 0.5–6.0 mg/mL. At least three concentrations for each sample condition were measured to ensure no concentration dependence. The ionic strength was set from 12 to 300 mM by varying the NaCl concentration. SAXS measurements were carried out using the BM29 beamline at the European synchrotron facility ESRF in Grenoble, France. The incident beam wavelength was 0.99 Å, the distance between the sample and the PILATUS 1M detector was set to 2864 mm, and the scattering vector range was 0.0036 Å^−1^ < q > 0.49 Å^−1^. The scattering vector was defined as $q=\frac{4\pi sin\theta }{\lambda }$, where 2θ is the scattering angle. For each sample and pure solvent, several successive frames (typically 10–25) of 1–2 s each, were recorded, enabling detection of radiation damage prior to further processing of the data. The background (pure solvent) was measured before and after the sample, and the average was subtracted from the signal from of the corresponding sample solution. The temperature was set to 20 ${}^{\circ }$C and I(0) was converted to absolute scale by measuring the scattering of water. The software Primus from the ATSAS package [33] was utilised for data inspection and analysis. The radius of gyration was determined for each sample by Guinier analysis, where $q\cdot R_{G}<=0.8$. The distance distribution functions were calculated using GNOM and used as input for the ab initio envelope reconstruction program DAMMIF [34]. Twenty independent reconstructions were completed using DAMMIF, the results were checked for consistency, averaged and filtered back to the volume of the original models. Fits of the calculated envelope to the scattering curve are shown in Supplementary Figure S4. The goodness fit of the experimental scattering curve to the computer simulation was determined by calculating $\chi^{2}$:

(1) $$\chi^{2}=\frac{1}{Np}\sum_{i=1}^{Np}{\left[\frac{I_{\exp}-I_{\mathrm{sim}}}{I_{\mathrm{sim}}}\right]}^{2},$$

where $N_{p}$ is the number of data points, $I_{\exp}$ is the experimental scattering data, and $I_{\mathrm{sim}}$ the simulated scattering data.

### 2.3. NMR

All NMR experiments were acquired on a Bruker Avance III 600 MHz (^1^H) spectrometer equipped with a TCI cryogenic probe. Pulsed Field Gradient (PGF) diffusion experiments were recorded on samples containing 1 mM Hst5 in 20 mM MES pH 6.7, 150 mM NaCl, 10% D_2_O, and 0.25% (*v/v*) 1,4-Dioxane at 20 ${}^{\circ }$C. In low pH experiments, the MES buffer was replaced with 20 mM sodium acetate pH 4.0. For experiments involving Zn^2^+, 4 mM ZnCl_2_ was added to the buffer. A standard Bruker PFG-longitudinal eddy current delay sequence with bipolar gradients and solvent pre-saturation during the relaxation delay of 3 s was used [31]. A total of 32 spectra with gradients strengths ranging from 2% to 98% of its maximum value were recorded. A diffusion time Δ of 200 ms and gradient length δ of 2 ms were used in all experiments. The pseudo 2D data was Fourier transformed and baseline corrected in Topspin (Bruker, Billerica, MA, USA), and subsequently analysed in Dynamics Center (Bruker). The diffusion constants were determined by fitting of peak intensity decays using the Stejskal-Tanner equation:

(2) $$I=I_{0}e^{-g^{2}\gamma^{2}\delta^{2}\left(\Delta -\frac{\delta }{3}\right)D}$$

where *I* is the intensity, *g* is the gradient strength, γ the gyromagnetic ratio of ^1^H, δ the gradient length, Δ the diffusion time, and *D* the diffusion constant. The diffusion constants from a selection of peaks in the aromatic and aliphatic side-chain regions were plotted as histograms and fitted to a Gaussian function using:

(3) $$f\left(x\right)=A\cdot e^{-\frac{{\left(x-\mu \right)}^{2}}{2\sigma^{2}}},$$

where μ is the mean, σ the standard deviation, and *A* is the amplitude. Different bin sizes where evaluated to check for robustness of the extracted parameters. 1,4-dioxane with a known hydrodynamic radius of 2.12 Å was used as an internal reference and used for the calculation of $R_{h}$ according to Wilkins et al. [35]:

(4) $$R_{h,\mathrm{prot}}=\frac{D_{\mathrm{ref}}}{D_{\mathrm{prot}}}\cdot R_{h,\mathrm{ref}},$$

### 2.4. Isothermal Titration Calorimetry

Isothermal titration calorimetry (ITC) experiments were carried out using a MicroCal ITC200 (GE Healthcare) at 20 ${}^{\circ }$C. Prior to each experiment, samples were centrifuged at 20.000 *g* for 10 min followed by degassing for 15 min. In each titration either, Hst5, Hst5_ZM_ or Hst5_DZM_ at concentrations ranging from 150–300 µM were placed in the cell, and ZnCl_2_ in the syringe at a concentration of 3.52 mM. The experiments were performed in 20 mM MES (pH 6.7), 150 mM NaCl. After an initial delay of 180 s, 18 injections of 2 µL ZnCl_2_ solution was titrated into Hst5 or variant, with each injection separated by 180 s. The heat of dilution was measured in control experiments without Hst5 and was subtracted from the raw data. The thermodynamic parameters were obtained by fitting the data to a single-site binding model within the Origin software (MicroCal, MA, USA). All experiments were performed in triplicates or more, and the average values are given with standard errors of the mean in Table 1.

### 2.5. Coarse-Grained Model

Previous studies completed in the Skepö group have shown that there is a good agreement between the Monte-Carlo approach and more detailed atomistic molecular dynamics simulations using Gromacs [26,36]. Despite the fact that the coarse grained approach provides a less detailed description, the main advantage of using this model is the ability to study intermolecular and multibody interactions. The individual residues of the proteins, i.e., the amino acids, were represented by hard spheres (beads) that mimic their excluded volume including the hydration layer, and were connected via harmonic bonds [14]. The N- and C-termini were explicitly included to account for the extra charge. The bead radius was set to 2 Å providing a realistic contact separation between the charges and an accurate Coulomb interaction. The non-bonded spheres interacted through a short-ranged attractive interaction as well as electrostatic interactions, where the interparticle electrostatic interactions were described on the Debye-Hückel level. The simulations were performed at constant pH with point charges. Each monomer was either charged, or neutral, depending on the amino acid sequence. The total potential energy of the simulated system contained bonded and non-bonded contributions, and was given by:

(5) $$U_{\mathrm{tot}}=U_{\mathrm{nonbond}}+U_{\mathrm{bond}}=U_{\mathrm{hs}}+U_{\mathrm{el}}+U_{\mathrm{short}}+U_{\mathrm{bond}},$$

where the non-bonded energy was assumed to be pairwise additive according to:

(6) $$U_{\mathrm{nonbond}}=\sum_{i<j}u_{\mathrm{ij}}\left(r_{\mathrm{ij}}\right),$$

where $r_{ij}=\left|R_{i}-R_{j}\right|$ is the center-to-center distance between two monomers, and *R* refers to the coordinate vector. The excluded volume was taken into account through the hard-sphere potential, $U_{hs}$, given by:

(7) $$U_{hs}=\sum_{i<j}u_{ij}^{hs}\left(r_{ij}\right),$$

which sums up over all amino acids. The hard-sphere potential between two monomers in the model was given by:

(8) $$u_{ij}^{hs}\left(r_{ij}\right)=\left\{\begin{array}{c} 0,\;r_{ij}\geq R_{i}+R_{J}\\ \infty ,\;r_{ij}<R_{i}+R_{J} \end{array}\right\}$$

where $R_{i}$ and $R_{j}$ denote the radii of the beads. The electrostatic potential was given by an extended Debye-Hückel potential according to:

(9) $$U_{el}=\sum_{ij}u_{ij}^{el}\left(r_{ij}\right)=\sum_{i<j}\frac{z_{i}z_{j}e^{2}}{4\epsilon_{0}\epsilon_{r}}\cdot \frac{exp\left[-\kappa \left(r_{ij}\left(R_{i}-R_{j}\right)\right)\right]}{\left(1+\kappa R_{i}\right)\left(1+\kappa R_{j}\right)}\cdot \frac{1}{r_{ij}},$$

where *e* is the elementary charge, κ denotes the inverse Debye screening length, $\epsilon_{0}$ is the vacuum permittivity, and $\epsilon_{r}$ the dielectric constant for water. The short-ranged attractive interaction between the monomers was included through an approximate arithmetic average over all amino acids, given by:

(10) $$U_{short}=-\sum_{i<j}\frac{\epsilon }{r_{ij}^{6}},$$

where ϵ reflects the polarisability of the proteins, and thus sets the strength of the interaction. In this model, ϵ was set to $0.6\cdot 10^{4}$ kJ Å${}^{6}/\mathrm{mol}$, giving an attractive potential of 0.6 kT at closest contact. The bonded interaction, a harmonic bond, was given by:

(11) $$U_{bond}=\sum_{i=1}^{N-1}\frac{k_{bond}}{2}\cdot {\left(r_{i,i+1}-r_{0}\right)}^{2},$$

where $r_{i,i+1}$ denotes the distance, in Å, between two connected monomers with the equilibrium separation $r_{0}=4.1$ Å. The force constant is set to $k_{bond}=0.4\;N/m$, whereas *N* denotes the number of monomers of the protein. The proteins were assumed to be totally flexible.

The self-association of the wild type (WT) peptide and of its variants upon addition of Zn^2^+ were modelled by adding a short-ranged interaction between the monomers that were expected to be involved in the association process, utilising Equation (10). The interaction strength, ϵ, was set to $3.0\cdot 10^{4}\;\mathrm{kJ}$ Å${}^{6}/\mathrm{mol}$, giving an attractive potential of 3.0 kT at closest contact.

### 2.6. Simulation Aspects

The equilibrium properties of the model systems were obtained by applying Monte Carlo simulations in the canonical (NVT) ensemble, that is, constant volume, number of particles, and temperature (T = 298 K), utilising the Metropolis algorithm. The protein chains were enclosed in a cubic box with a box length at least 30 times longer than the radius of gyration of the protein. Periodic boundary conditions were applied in all directions. The long-ranged Coulomb interactions were truncated using the minimum image convention. Four different types of displacements were allowed: (i) translational displacement of a single bead, (ii) pivot rotation, (iii) translation of the entire chain, and (iv) slithering move, in order to accelerate the examination of the configurational space [32]. The probability of the different trial moves was weighted to enable single-particle moves 20 times more often than the other three. Initially, the protein was randomly placed in the box and an equilibrium simulation of typical $2\cdot 10^{5}$ trial moves/bead was performed, whereas the proceeding production run comprised $2\cdot 10^{6}$ passes divided into ten subdivisions. The acceptance ratio in the simulations added up to a total of approximately 30%. The radius of gyration and the end-to-end distance probability distribution functions of the proteins i.e., the conformational ensembles, were analysed to confirm that the simulations were sampled accurately. The reported uncertainty of simulated quantities is one standard deviation of the mean. It is estimated from the deviation among the means of the subdivisions of the total number of MC passes according to:

(12) $$\sigma^{2}\left(\langle x\rangle \right)=\frac{1}{n_{s}\left(n_{s}-1\right)}\cdot \sum_{s=1}^{n_{s}}{\left({\langle x\rangle }_{s}-\langle x\rangle \right)}^{2},$$

where ${\langle x\rangle }_{s}$ is the average of quantity *x* from one subdivision, 〈x〉 the average of *x* from the total simulation, and $n_{s}$ the number of subdivisions used which here is 10. The simulations were performed by using the integrated Monte Carlo/molecular dynamics/Brownian dynamics simulation package Molsim [37].

### 2.7. Structural Analysis

The model was validated by comparing the simulated scattering intensities with the experimental scattering intensities obtained by SAXS. For a system containing *N* identical scattering objects, the structure factor is given by:

(13) $$S\left(q\right)=\langle \frac{1}{n}{\left|\sum_{j=1}^{N}exp\left(iq\cdot r_{j}\right)\right|}^{2}\rangle .$$

The total structure factor can further be decomposed into partial structure factors given by:

(14) $$s_{ij}\left(q\right)=\langle \frac{1}{{\left(N_{i}N_{j}\right)}^{\frac{1}{2}}}\cdot \left[\sum_{i=1}^{N_{i}}exp\left(iq\cdot r_{i}\right)\right]\left[\sum_{j=1}^{N_{j}}exp\left(iq\cdot r_{j}\right)\right]\rangle .$$

The total and partial S(q) are related through:

(15) $$s\left(q\right)=\sum_{i=1}^{N_{i}}\sum_{j=1}^{N_{j}}\frac{{\left(N_{i}N_{j}\right)}^{\frac{1}{2}}}{N}\cdot S_{ij}\left(q\right).$$

For a point scatterer the form factor is constant, inferring that the scattering intensity is proportional to the structure factor. In order to account for an approximate effective particle/residue form factor, the scattering profile further needs an appropriate normalisation, such that $I_{0}$ coincides with the experimental scattering profile.

The average oligomer size as well as the oligomer size distribution were determined using a geometrical definition, and we considered them as clustered if the distance between at least one amino acid from one protein did not exceed 6 Å from another protein. The formation of an oligomer from the SAXS-experiments have been determined by defining the ratio β, which corresponds to the forward scattering, $I_{0,Zn}$, normalised by the concentration for the protein clustered with Zn^2^+, $C_{cp,Zn}$, divided by the forward scattering normalised with the protein concentration in the vicinity of Zn^2^+ i.e., $I_{0}$ and $c_{p}$ respectively, see Equation (16).

(16) $$\beta =\frac{I_{0,Zn}/C_{cp,Zn}}{I_{0}/C_{p}}.$$

Thus, β=1 indicates no oligomer formation, whereas β>1 does.

## 3. Results And Discussion

**Figure 3:**
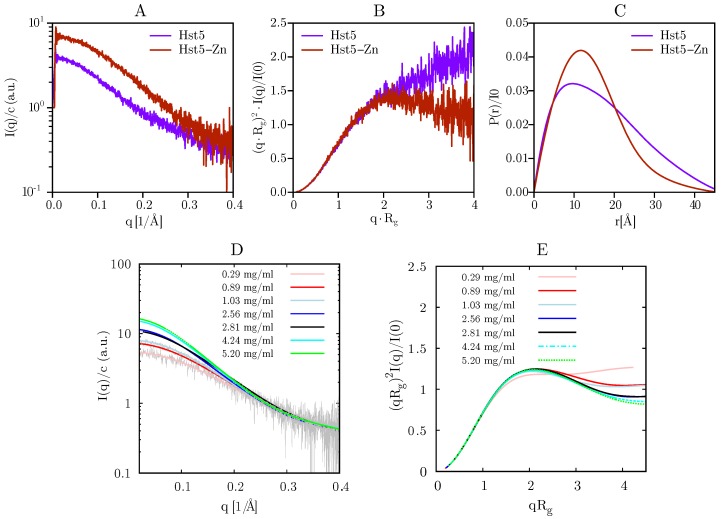
**SAXS analysis of Hst5 in the absence and presence of ZnCl_2_.** (**A**) Comparison of the intensity function normalised by concentration for 0.9 mg/mL Hst5, in 20 mM MES-buffer, pH 6.7, 150 mM NaCl and 4 mM ZnCl_2_. (**B**) SAXS data shown as a dimensionless Kratky plot. (**C**) Plot of the intra-peptide distance distribution determined by indirect Fourier transform, for Hst5, with either NaCl (purple curves) or with ZnCl_2_ (red curves). (**D**,**E**) Concentration dependent SAXS-measurements of Hst5 in the presence of ZnCl_2_, showing the intensity curve normalised with protein concentration and the corresponding Kratky plot.

We have earlier shown that the form factor of Hst5 under physiological conditions can be obtained at a concentration of 1 mg/mL, and that its solution behaviour is consistent with that of an IDP [25,26]. To investigate how the addition of Zn^2^+ to the histidine-rich protein changes its properties, we first employed SAXS. In the absence of Zn^2^+, Hst5 is a monomer with an estimated MW of 2.9±0.2 kDa based on the normalised intensity function (Figure 3A). Addition of Zn^2^+ resulted in an increase of I(0) corresponding to an increase in the measured molecular mass to 4.8±0.4kDa. Addition of Zn^2^+ furthermore led to a compaction of the overall protein as shown by the downward curvature in the Kratky plot (Figure 3B). The compaction was also supported by a redistribution of the pair distance distribution P(r) towards shorter distances in the protein, although no effect was observed on the maximum interparticle distance of the chain (Figure 3C).

Investigation of the concentration dependence of the SAXS profiles as seen in Figure 3D showed that the mass-weighted distribution was affected by the protein concentration, and that the complexes increased in size with increasing protein concentration. Furthermore, precipitation was observed for high ratios of Zn^2^+:protein. The dimensionless Kratky plot (Figure 3E) showed that the oligomers obtained a more compact shape when compared to the random coil formation for the monomer, which was clearly visible for Hst5 at concentrations of 0.29 mg/mL. Furthermore, a plateau value was reached at a peptide concentration of around 5 mg/mL.

**Figure 4:**
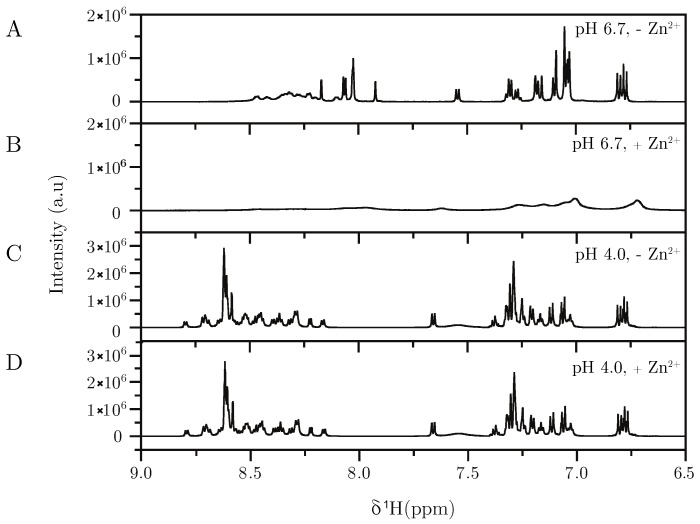
**^1^H 1D NMR spectra of the aromatic region of Hst5 under different conditions**. (**A**) pH 6.7, 150 mM NaCl, (**B**) pH 6.7, 150 mM NaCl, 4 mM ZnCl_2_ (**C**) pH 4.0, 150 mM NaCl and (**D**) pH 4.0, 150 mM NaCl, 4 mM ZnCl_2_. All spectra were recorded at 20 ${}^{\circ }$C.

To substantiate our observations further, we performed 1D ^1^H NMR measurements on Hst5 in the presence and absence of ZnCl_2_ (Figure 4 and Supplementary Figures S1 and S5). At pH 6.7, the spectrum of Hst5 showed sharp lines and was relatively well-resolved (Figure 4A). In contrast, addition of 4x molar excess of ZnCl_2_ caused severe line broadening, which could be due to formation of oligomers, and thus a decrease in tumbling rate (Figure 4B). However, our SAXS data suggested only a three-fold increase in molecular weight, which is not sufficient to induce this degree of broadening. Thus, chemical exchange between different unbound/bound states at a rate similar to the difference in resonance frequency between the different states is more likely to be the origin. Hst5 contains a high number of histidines known to coordinate Zn^2^+. Their involvement in binding is supported by the fact that no line broadening was observed upon addition of Zn^2^+ at pH 4, where the histidines are protonated (Figure 4C,D). This observation is in line with SAXS experiments at pH 4, which did not indicate any oligomerisation (data not shown). Hence, it is possible that Zn^2^+ binding does not require specific histidine residues or a specific structure, but shuffles between different binding sites in a highly dynamic manner, resulting in the observed line broadening.

### 3.1. Tracing the Interaction Through Variants

To achieve a more in-depth understanding of the oligomerisation and Zn^2^+ binding to Hst5, and to address the possibility of dynamic Zn^2^+ coordination, we designed variants of Hst5, where we modulated the number and position of the histidine residues. In this process, we noted that Hst5 contains a zinc-binding-like motif, HAKRHH, in its N-terminal, and we therefore mutated this orthogonal to the known motif. Additionally, we randomised the sequence of Hst5, leading to a total of five different variants. For substitutions of the histidines, we chose glutamine with similar size, atomistic chemical skeleton, and hydrogen bonding potential as histidine, and thus can be viewed as a pH-insensitive histidine-analogue. The five variants, and their primary structures, are listed in Figure 2.

**Figure 5:**
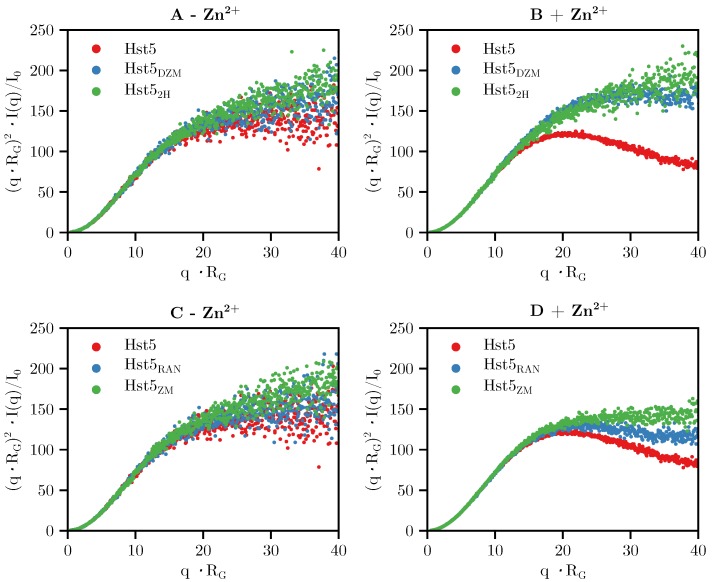
**Kratky plots visualising the difference in shape between Hst5 in the absence and presence of Zn^2^+.** (**A**,**B**) shows Hst5, Hst5_DZM_, and Hst5_2H_ in the absence and presence of Zn^2^+, respectively. (**C**,**D**) shows Hst5, Hst5_RAN_, and Hst5_ZM_ in the absence and presence of Zn^2^+, respectively.

Using SAXS and NMR, the conformational properties of WT Hst5 and the five variants were measured and compared (Table 2). The Kratky plots, Figure 5, revealed that in the absence of Zn^2^+, the WT Hst5 and variants obtained the same overall shape. This was confirmed by NMR diffusion experiments, which showed a size of R_h_ of approximately 13 Å for all variants. Furthermore in line with data published by Henriques et al. [38], the R_g_\R_h_ ratio of ≈ 1 suggests that the solvation layer around IDPs can be considered to be very small in comparison to structured proteins. The radii of gyration obtained from SAXS indicated a minor effect on the extension of the peptides when changing the order of the histidines as in Hst5_RAN_, but it was difficult to find a correlation and an underlying mechanism. No correlation between the R_h_ (or the R_g_) with the number of histidines was seen. We did observe an overall correlation between the theoretical the R_g_/R_h_ ratio as was calculated from the empirical relation published previously [39] and the current data (supplementary Figure S6). Investigation of the overall shape of Hst5 by inspection of the Kratky plots of the variants indicated that removal of the zinc-motif (Hst5_DZM_) and/or the zinc-binding-like motif (Hst5_ZM_\Hst5_2H_), diminished the effects of Zn^2^+ on compaction (Figure 5), highlighting that the presence of both histidine motifs is vital for this effect. Randomising the sequence also slightly reduced the compaction of Hst5 in the presence of Zn^2^+, suggesting that not only the amount of histidines, but also their relative position is important for compaction. Interestingly, we could not detect any compaction of WT Hst5 nor or of Hst5_RAN_ using NMR, likely due to the extensive line broadening observed upon addition of Zn^2^+, deeming the compacted conformation of Hst5 NMR invisible.

**Figure 6:**
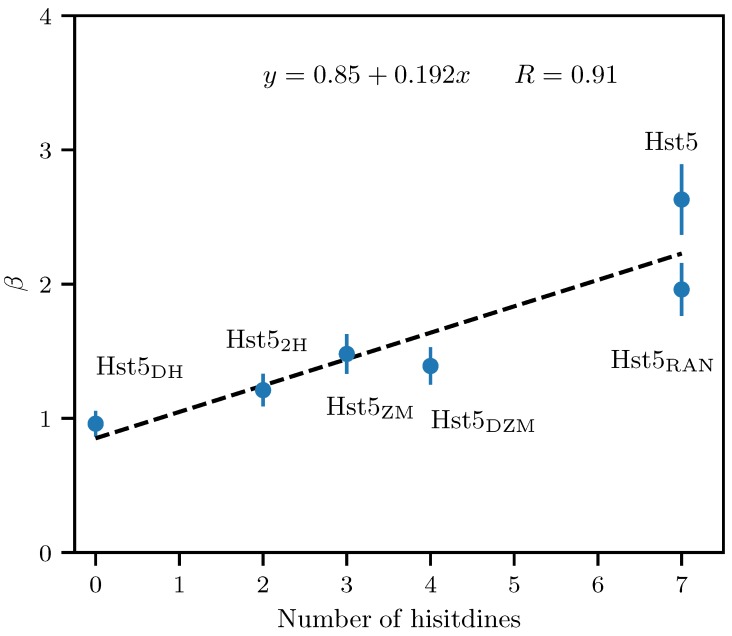
**Correlation between the number of histidines and oligomerisation of Hst5 in the presence of Zn^2^+**. The mass increase of the system defined as, β, see Equation (16), as a function of the number of histidines. Here β = 1.0 indicates no mass increase in the system i.e., no oligomerisation, whereas β > 1 does.

The zinc-induced oligomerisation observed for WT Hst5 was evaluated by comparing the forward scattering of the system in the absence and in the presence of Zn^2^+, normalised to the concentrations as,$\frac{I_{0,zn}/c_{p,Zn}}{I_{0}/c_{p}}$; from now on denoted β. Here β = 1.0 indicates no mass increase in the system i.e., no oligomerisation, whereas β > 1 does. Comparing the β-values for WT Hst5 and Hst_RAN_ (Table 2) showed that randomising the sequence decreased β from ≈ 2.5 to 2, but did not abolish oligomerisation. The effect of keeping the HExxH-motif and removing the histidines of the zinc-binding-like motif (Hst5_ZM_) or vice versa (Hst5_DZM_), affected the oligomerisation to the same extent (β ≈ 1.4–1.5) in agreement with the NMR diffusion experiments (14.2±0.4 Å and 16.2±0.9 Å for Hst5_DZM_ and Hst5_ZM_, respectively). These results suggest that while the order of the histidines may have a small effect on oligomerisation, it is the number of histidines that is most important. In line with this, no binding was detected by NMR (supporting information Figure S1), and thus no oligomerisation (β ≈ 1) occurred for Hst5_DH_ in which all histidines are removed. Accordingly, if only two histidines remained in the sequence, Hst5_2H_, oligomerisation occurred only to a very small extent (β ≈ 1.2). Thus, a correlation between oligomerisation given by β, and the number of histidines, reveal a linear relationship (Figure 6). Based on this we conclude that the order of histidines plays a less important role in zinc binding and oligomerisation, whereas the number of histidines in the protein sequence regulates the process.

**Figure 7:**
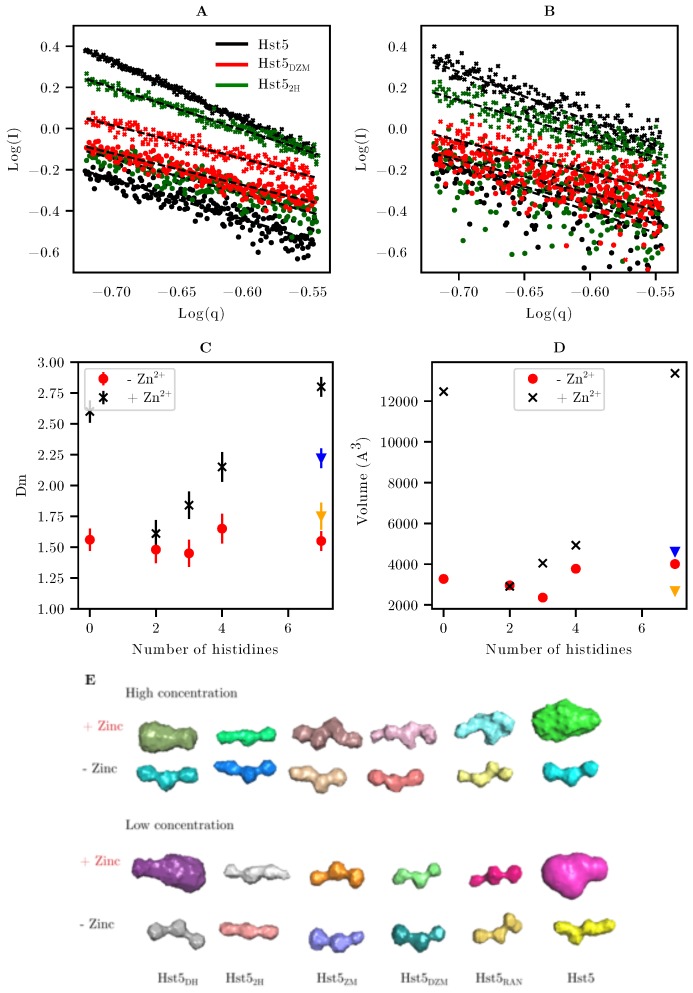
**The effect of Zn^2^+ on the shape of Hst5 and selected variants** (**A**) Log-Log plot of SAXS of 5 mg/mL Hst5 variants in the absence of Zn^2^+ (circles) and presence of Zn^2^+ (squares). The dashed lines represent a fit of the power law relationship to the scattering data from which the fractal dimension D_m_ was estimated. (**B**) The same plot as A shown for low concentration scattering data (1 mg/mL). (**C**) Plot of D_m_ against the number of histidines present in the Hst5 variants in the absence of Zn^2^+ (red circles) and in the presence of Zn^2^+ (black crosses). (**D**) The volume of the scattering envelope, in the presence and absence of Zn^2^+. The randomised sequence is shown as triangles where yellow is in the absence of Zn^2^+ and blue in the presence. (**E**) Scattering envelopes produced by DAMMIF corresponding to the volumes shown in Figure 7D.

Since there were different degrees of mass increase corresponding to different sizes of oligomers formed in the different variants, this hampered the analyses of shape and R_g_’s. Even though Hst5 is a short peptide, which decreases the probability to attain a large conformational ensemble, the variants generally possessed a more extended shape than Zn^2^+ bound Hst5, as seen in the Kratky plots in Figure 5. From the experimentally determined β-value, we noticed that upon addition of Zn^2^+, oligomers were formed when at least two histidines were present. Analysing the Kratky plot showed that Zn^2^+ addition resulted in a more compact shape, compared to a random coil (see Supplementary Figure S2). Chain expansion or collapse can be probed by SAXS by analysing the fractal dimension at intermediate q-range. In this region the intensity has a power law dependence on q, thus, by plotting log(I) against log(q), the mass fractal dimension, D_m_, can be determined [40]. Unlike R_g_ and R_h_, D_m_ is sensitive to the favourable or unfavourable solvation of the measured chains. Larger values of D_m_ reflect unfavourable interactions with the solvent, and more compact conformations. Figure 7A,B shows the linear fit for this region of the SAXS data for Hst5, as well as of the variants with a reduced number of histidines, for both concentrations measured. We note that although this region is expected to exist, the noise can be quite high and thus, we focused only on the high concentration measurements (values of D_m_ obtained for low concentrations samples are shown in Supplementary Figure S3B,D). In the absence of Zn^2^+, the measured D_m_ was invariant with the number of histidines. The estimated D_m_ of about 1.6 observed for all variants, (Figure 7C), was close to the expected value of a well solvated polymer (1.7), and agreed well with the shape observed in the Kratky plot [40]. Upon addition of Zn^2^+, D_m_ increased for all of the Hst5 variants. This increase could be caused by either a collapse of the polypeptide chain or the formation of oligomers. In the low concentration measurements, the observed differences in D_m_ were primarily observable in WT Hst5, and some effects were noticed for the randomised sequence. The increase is linear with the number of histidines present in the sequence apart from His5_RAN_ and His5_DH_. It is therefore more likely that the increase in D_m_ corresponds to oligomer conformations of Hst5, which are less exposed to solvent [40]. To confirm this, the average scattering envelopes were calculated from the P(r) functions using DAMMIF, see models in Figure 7E.

Typically for IDPs, this approach does not provide detailed information as the scattering from a heterogeneous population yields an average of the entire population. However, we note that an increase in the apparent scattering envelope would correspond to an increase in the average protein size, caused by the contribution of an oligomeric population. Figure 7D shows the effects of Zn^2^+ on the volume of the calculated scattering envelopes from different variants. The change in volume follows the same trend as the change observed in the fractal dimension, hence we conclude that the observed change in D_m_ is caused by the presence of oligomers, which have a more compact conformation. A large increase in the volume of the scattering envelope was observed in WT Hst5 upon addition of ZnCl_2_. This supports our previous observation suggesting that the presence of oligomerisation is mediated by histidine-zinc interactions. This ability is increased when the histidine residues are organised within a zinc-binding motif. Interestingly, the variant Hst5_DH_ also became more compact when Zn^2^+ was added to the solution as observed in the D_m_ values, and the increased volume. This is also visible in R_g_, which decreased from 13.9 => 13.4 Å.

### 3.2. Comparison with Computer Simulation

**Figure 8:**
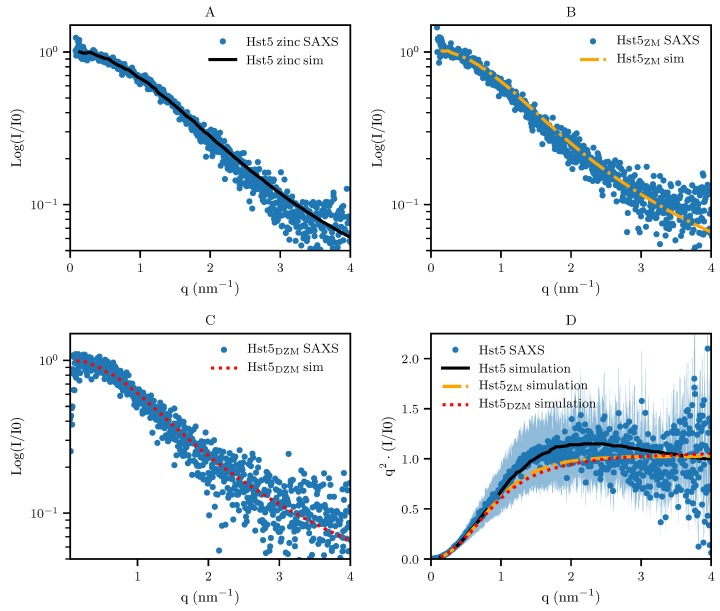
**Comparison of experimental SAXS curves with those determined by computer simulation for WT Hst5**. The intensity function normalised by concentration, I(q)/c, and the corresponding Kratky plot, $q^{2}I\left(q\right)/I\left(0\right)$, for SAXS of Hst5 and Monte Carlo simulations, where Figures (**A**–**C**) depict a comparison between the experimental SAXS-spectra of Hst5/variants and simulations, where the additional short-ranged attractive interactions is taken into consideration. The $\chi^{2}$ of the fits to the data are as follows (**A**) 2.2 (**B**) 1.9 and (**C**) 1.7. Figure (**D**) shows the SAXS data as a Kratky plot. Colours as in (**A**–**C**). The data given in the Figure corresponds to a peptide concentration of ≈ 1 mg/mL.

**Figure 9:**
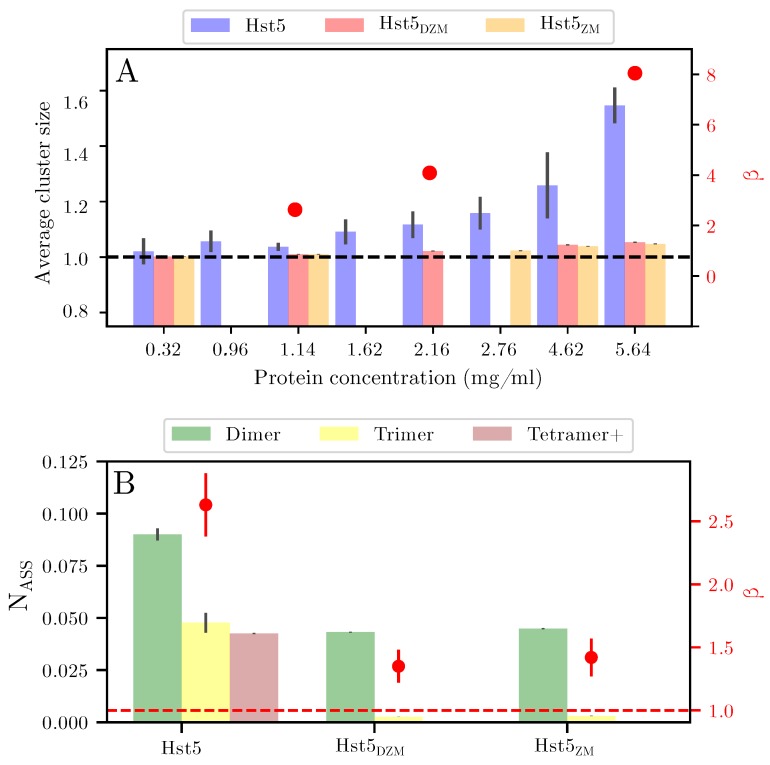
**Distribution of oligomers determined by computer simulation for Hst5 and variants**. (**A**) Average cluster size for Hst5 and variants as indicated determined by computer simulation. Shown for increasing concentrations of the respective protein. The scatter points correspond to the β values determined for Hst5 from SAXS (red y-axis). (**B**) Distribution of the formed clusters obtained by computer simulation, the bar chart corresponds to the determined N_ass_. Tetramer + corresponds to tetrameric and higher oligomers. The scatter points correspond to the β values determined from SAXS (red y-axis, as shown in Figure 7). The red and black dashed lines represents a β value of 1, i.e., a monomeric protein.

From the above experimental results it is plausible that the mechanism of Aβ oligomer formation as proposed by Polshakov et al. [20] is applicable to Zn^2^+-coordination by Hst5. To address this, we used our established coarse-grained model and Monte Carlo simulations with a slight modification, that is, a short-ranged attractive interaction decaying as $1/r^{6}$ was implemented among the histidines and the Zn^2^+-binding motif. In the simulations, the experimental concentrations were mimicked, hence multibody interactions were taken into account. For example, a simulation box of 450 Å in X, Y, and Z-directions, and a protein concentration of 1 mg/mL, corresponds to approximately 16 chains. The scattering intensity curves for Hst5_ZM_, Hst5_DZM_, and Hst5 (Figure 8A–C, respectively) were modelled by applying a recent protocol [21], and compared to the experimental scattering data. There was good agreement between the model and the experimental data ($\chi^{2}$) for Hst5 + Zn^2^+≈2.2. This correspondence was also evident for the shape of the peptides, as visualised in the Kratky plot in Figure 8D. The number average of the formed oligomers as a function of peptide concentration was further analysed by simulations. As was shown in Table 2, β for Hst5_DZM_ and Hst5_ZM_ were similar in size i.e., 1.4–1.5, whereas for Hst5 it was ≈ 2.5. From the simulations, the average oligomer size was extracted (Figure 9A), supporting these observations and providing additional support that both the HExxH-motif as well as the second histidine binding motif are needed for oligomer formation, and that the most frequent average cluster size was between monomer and dimer.

The oligomers were further investigated by analysing the distribution of the formed clusters, which gave an indication of the largest observed cluster (see Figure 9B). For a concentration of 2.8 mg/mL Hst5, there was >1% probability for pentamer formation, where the main part corresponded to monomers (82%) and dimers (9%). For the variants Hst5_DZM_ and Hst5_ZM_, the main contribution came from monomers (95%), with approximately 4% of dimers. Hence, this once again confirms that the cooperation of histidines play an important role for the formation of clusters, their average size, and their polydispersity. Furthermore, considering the small population of oligomers, these results suggest that the compaction observed occurs in the monomer. As was stated above, the size of the oligomers was dependent on the peptide concentration, which was also the case for the polydispersity. For example, simulation of approximately 1 mg/mL Hst5 gives 94% monomers, 5.5% dimers, and 0.5% trimers. There was a slight but very low probability that oligomers of the size of pentamers formed. Comparison with the β value obtained by SAXS showed a good agreement with the oligomer distributions obtained by computer simulation (Figure 9). Thus, in conclusion, we find that Hst5 forms dynamic oligomers in the presence of Zn^2^+ in which a compact monomer dominates, followed by a concentration dependent increase in the size of oligomers, with the dimer being the second most dominant species.

### 3.3. Stoichiometry, Coordination And Thermodynamics

**Figure 10:**
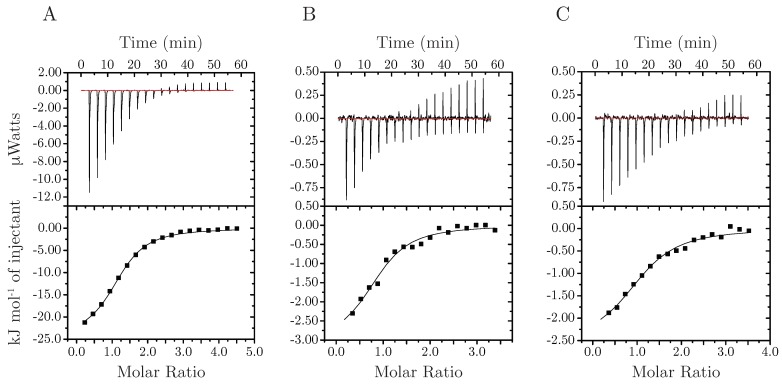
**ITC measurements of interactions between Hst5 variants and Zn^2^+**. Plots showing ITC data representative of the experiments. Titration of Zn^2^+ with (**A**) WT Hst5, (**B**) Hst5_DZM_, and (**C**) Hst5_ZM_.

To understand the thermodynamics as well as mapping the stoichiometry of Zn^2^+-binding to Hst5, we employed isothermal titration calorimetry, Figure 10, and included the two variants where each of the zinc-binding motifs were knocked out, Hst5_DZM_ and Hst5_ZM_ (Table 1). The WT Hst5 bound Zn^2^+ with µM affinity (Kd = 26 µM) and was mainly enthalpically driven with a minor entropic contribution. Despite the two potential Zn^2^+ binding sites in WT Hst5, the binding stoichiometry for the interaction with Zn^2^+ was one suggesting either 1:1 or 2:2 binding. The simulations suggested a very small oligomer population making a 1:1 binding or a mix between 1:1 and 2:2 binding between Hst5 and Zn^2^+ most likely. In solution Zn^2^+ is most often octahedrally coordinated to water, however, in protein complexes, tetrahedral coordination is by far most dominant [41,42]. In structural stabilisation, four protein ligands are most common whereas in many enzymes three protein ligands in conjunction with water or some other solvent molecule prevails [41,42,43]. Despite almost equal presence of histidines and cysteines in Zn^2^+ coordination, no structures with four coordinating histidines (His_4_) were reported by Laitaoja et al. [41] Instead histidines often coordinate Zn^2^+ in combination with an acidic residue (i.e., glutamate or aspartate), whereas Cys_4_ coordination is most common in several protein classes [41]. Despite this, His_4_ coordination has previously been reported for Aβ where it is responsible for inter-peptide aggregation [44]. While it is likely that His_4_ coordination plays a similar role for Hst5 oligomerisation, it is possible that other residues than histidine such as the glutamate from the HEXXH motif are involved in the compaction of Hst5, thereby contributing to the observed line broadening. The fact that we see no dual Zn^2^+ binding to Hst5 likely reflects the fact that it is more energetically favourable for Zn^2^+ to be coordinated by four histidines rather than three histidines and a water molecule [45]. Interestingly the two single motif variants Hst5_DZM_ and Hst5_ZM_ both bound Zn^2^+ with an affinity similar to that of WT Hst5. While this may not be surprising given the similarity of the two motifs, the thermodynamic profile of the two types of interactions were very different, whereas the WT Hst5 interaction was mainly driven by enthalpic contributions, the interactions with the two single motif variants were driven by a large entropic contribution likely caused by desolvation and release of counter-ions. While the same desolvation occurs in the case of WT Hst5, the compaction resulting in less rotational freedom for the WT Hst5 termini, bears a large entropic cost. Furthermore, the compaction of WT Hst5 likely also induces a number of inter-molecular interactions that are not formed in the single motif variants, perhaps involving the two aromatic residues between the motifs, thus explaining the difference in enthalpic contribution in the two types of interactions.

## 4. Conclusions

### Underlying Mechanism of The Oligomerisation

**Figure 11:**
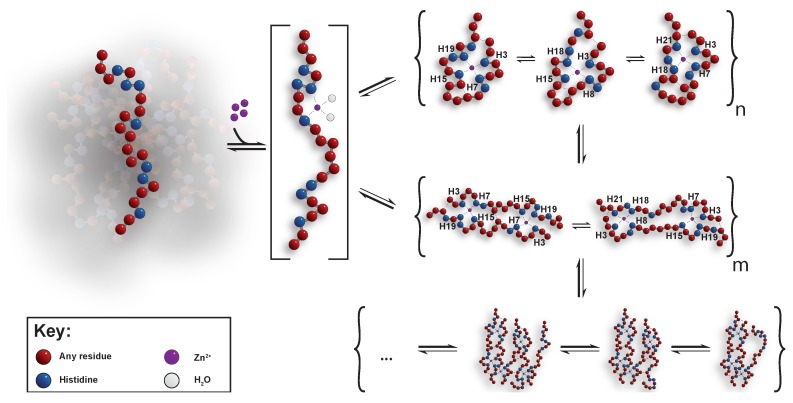
Schematic depiction of the mechanism of Zn^2^+ induced oligomerisation of Hst5.

The combined results obtained from SAXS, NMR, calorimetry, and simulations jointly support a coherent mechanism for how Zn^2^+ mediates oligomer formation of Hst5. Initially, Zn^2^+ can bind to any of the two binding motifs forming a one-to-one complex with similar affinity, as seen in the two variants Hst5_DZM_ and Hst5_ZM_. Likely, in a 1:1 complex with WT Hst5, Zn^2^+ would be coordinated by histidines from both motifs, leading to compaction. Once Zn^2^+ is bound, and the concentration increases, dimers are formed across two Hst5 chains, maintaining the 1:1 (2:2) stoichiometry, but with the possibility for more than one distinct coordination, giving rise to the observed severe line broadening. These dimers can then act as seeds and through interactions via networks of histidine:Zn^2^+ coordination, form higher ordered oligomers, although at a much lower population. This is in line with the suggested mechanism of oligomerisation of the Aβ1-16 region [20]. Hence it seems plausible that the dimerisation as well as the oligomerisation of Hst5 follows the same underlying mechanism as that proposed for the Aβ peptide [29] i.e., the initial binding between Hst5 and Zn^2^+ for the single peptide is through the residues H7/H8 and the amino acids in the zinc motif, E16, H18, and H19. Thereafter dimers are formed through coordination of Zn^2^+ between E16 and H19 on respective peptide, whereas finally oligomers are formed by dynamic coordination of the dimers through the histidines H7/H8 and H18, see Figure 11.

By entropic reasons, one can expect that it is the middle region of Hst5 that constitutes the preferred seed, since it will be more favourable for the system if the peptide ends remain unrestricted. This is supported by the thermodynamics measurements, as we see a much lower entropic penalty when either of the two motifs are mutated, whereas once both motifs are operational, a large entropic cost in Zn^2^+ binding is measured, compensated by much larger enthalpic gain. This mechanism i.e., that all histidines are involved in the dimerisation and oligomerisation process, is also in line with what Blindauer et al. [46] have seen for the histidine-rich glycoprotein HRGP330. By using NMR, they noticed a uniform line broadening of all histidine sidechains and most backbone resonances upon addition of a single molar equivalent of Zn^2^+, suggesting that there is no clear preferential biding site, and that the system contains several species in exchange on an intermediate NMR time scale. Furthermore, at high stoichiometric ratios of Zn^2^+/HRGP330, precipitation occurred, in line with our observations for Hst5. Furthermore, it has been suggested that the mechanism by which Zn^2^+ is expected to induce the formation of oligomers is via coordination to the N1 atom of the imidazole ring of the histidine, which causes cross-linking of the peptide through intermolecular His(N1)-Zn^2^+-His(N1) bridges [47]. This also provides the possibility for a dynamic coordination process, which as such might explain the line-broadening visible in the NMR experiments of Hst5. Finally, the dynamic interchange between different complexes with similar affinity allows Hst5 to act as a metal-ion buffering system in the mouth, where release and binding can be controlled thermodynamically, without leading to severe, and potentially toxic aggregation.

## Figures and Tables

**Table 1:** Thermodynamic analysis of Zn^2^+ binding to Hst5 and variants. All experiments were performed as described under “Experimental Procedures” and represent the average of three independent experiments.

	K_d_ (µM )	N	ΔH (kJ/mol)	−TΔS (kJ/mol)	ΔG (kJ/mol)
**Hst5**	26±1	1.18±0.01	−23.8±0.3	−2.0±0.3	−25.8±0.1
**Hst5_DZM_**	45±9	0.89±0.06	−3.1±0.3	−21.3±0.5	−24.4±0.5
**Hst5_ZM_**	61±10	0.94±0.04	−2.6±0.2	−21.1±0.4	−23.7±0.4

**Table:** (**a**)

	**c_p_(mg/mL)**	**c_p,Zn_(mg/mL)**	**R_g_(Å)**	**R_g,zn_(Å)**	**I_0_**	**I_0,Zn_**	**N_H_**	β
**Hst5**	1.01	1.03	13.4±0.01	11.5±0.02	3.07±0.036	8.22±0.041	7	2.63
**Hst5_RAN_**	0.94	0.94	11.9±0.06	11.1±0.09	3.47±0.062	6.80±0.062	7	1.96
**Hst5_DZM_**	0.92	1.09	12.1±0.07	11.5±0.08	2.99±0.066	4.91±0.031	4	1.39
**Hst5_ZM_**	1.13	1.23	12.8±0.05	13.6±0.02	3.12±0.0052	5.24±0.034	3	1.48
**Hst5_2H_**	1.27	1.10	14.1±0.04	12.9±0.05	3.25±0.048	3.44±0.057	2	1.21
**Hst5_DH_**	1.08	1.07	13.9±0.13	13.4±0.05	3.45±0.039	3.27±0.056	0	0.96

**Table:** (**b**)

	**N_H_**	**R_h_ (Å)**	**R_h,Zn_ (Å)**
**Hst5**	7	12.9 ± 0.7	12.9 ± 0.3 ^a^
**Hst5_RAN_**	7	12.9 ± 0.5	12.9 ± 0.3 ^a^
**Hst5_DZM_**	4	13.1 ± 0.4	14.2 ± 0.4 ^b^
**Hst5_ZM_**	3	13 ± 0.2	16.2 ± 0.9 ^b^
**Hst5_2H_**	2	13 ± 0.2	13.8 ± 0.4
**Hst5_DH_**	0	12.6 ± 0.6	13.3 ± 0.2

^a^ Extensive line broadening in the NMR spectra; ^b^ Light line broadening in the NMR spectra.

## Supplementary Materials

The following are available online at https://www.mdpi.com/2218-273X/9/5/168/s1. Supplemental Figure S1: 1H 1D NMR spectra of the aromatic region of Hst5 under different conditions and variants. Supplemental Figure S2: Kratky plots visualising the difference in shape between Hst5 in the absence and presence of zinc. Supplemental Figure S3: The effect of zinc on the shape of Histatin 5 and selected variants. Supplemental Figure S4: Fits calculated from the DAMMIF scattering envelope shown for each measured sample. Supplemental Figure S5: Representative diffusion data measured on Wt Hst5 A) with and B) without Zn2+ added. Supplemental Figure S6: Experimental Rg/Rh ratio compared with theoretical Rg/Rh ratio for the Hst5 variants.

## Abbreviations

The following abbreviations are used in this manuscript:

MDPI	Multidisciplinary Digital Publishing Institute
DOAJ	Directory of open access journals
TLA	Three letter acronym
LD	linear dichroism
