# Supplementary material for: Dynamical Oligomerisation of Histidine Rich Intrinsically Disordered Proteins Is Regulated through Zinc-Histidine Interactions
Source: Biomolecules. 2019 Apr 30;9(5):168. doi: 10.3390/biom9050168 (PMC6571702; doi:10.3390/biom9050168)
Supplement: Supplementary file 1 [file biomolecules-09-00168-s001.pdf]

Dynamical oligomerisation of histidine rich intrinsically disordered proteins is regulated through zinc-histidine interactions

Carolina Cragnell, Lasse Staby, Samuel Lenton, Birthe B. Kragelund, and Marie Skepö*

E-mail: marie.skepo@teokem.lund.se

Supplementary information

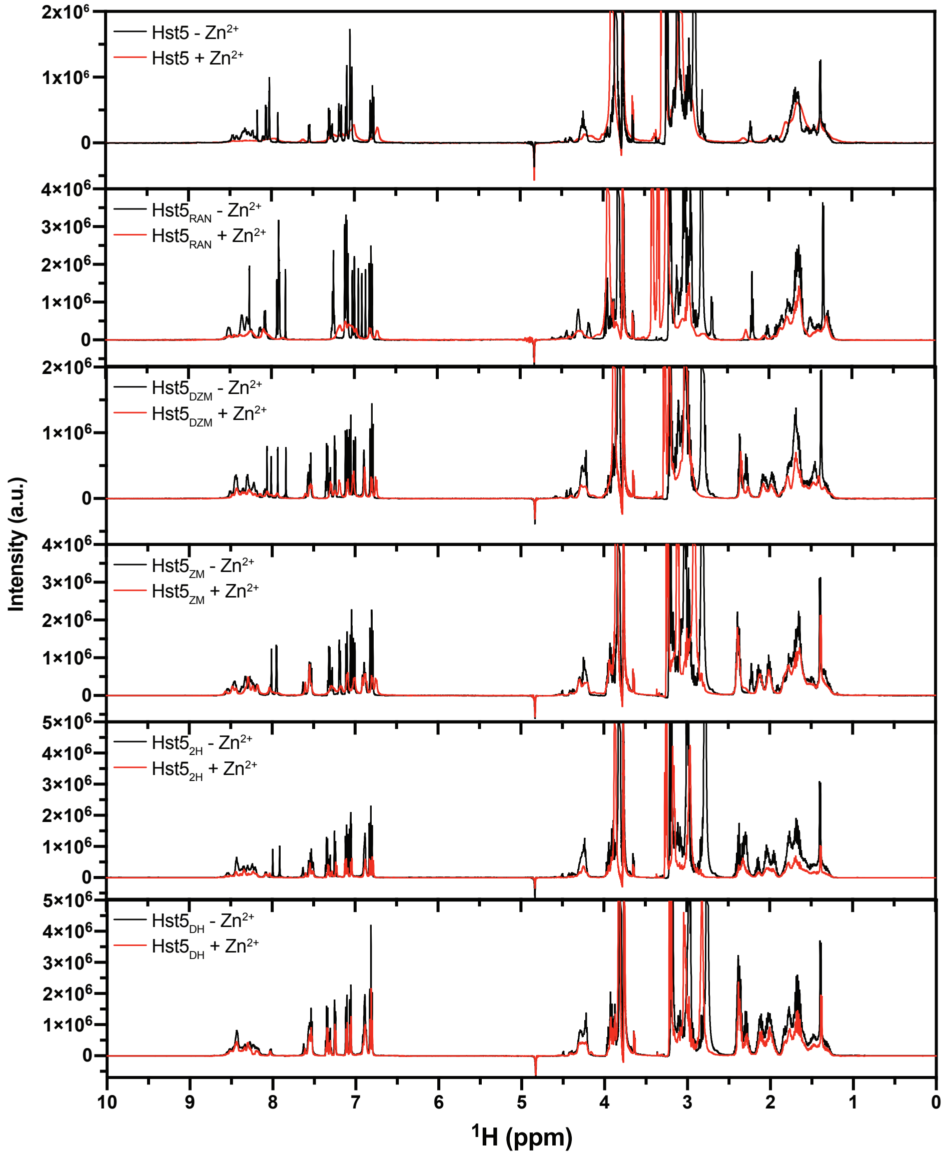

Supplementary Figure 1: ^{1}H 1D NMR spectra of Hst5 and variants.

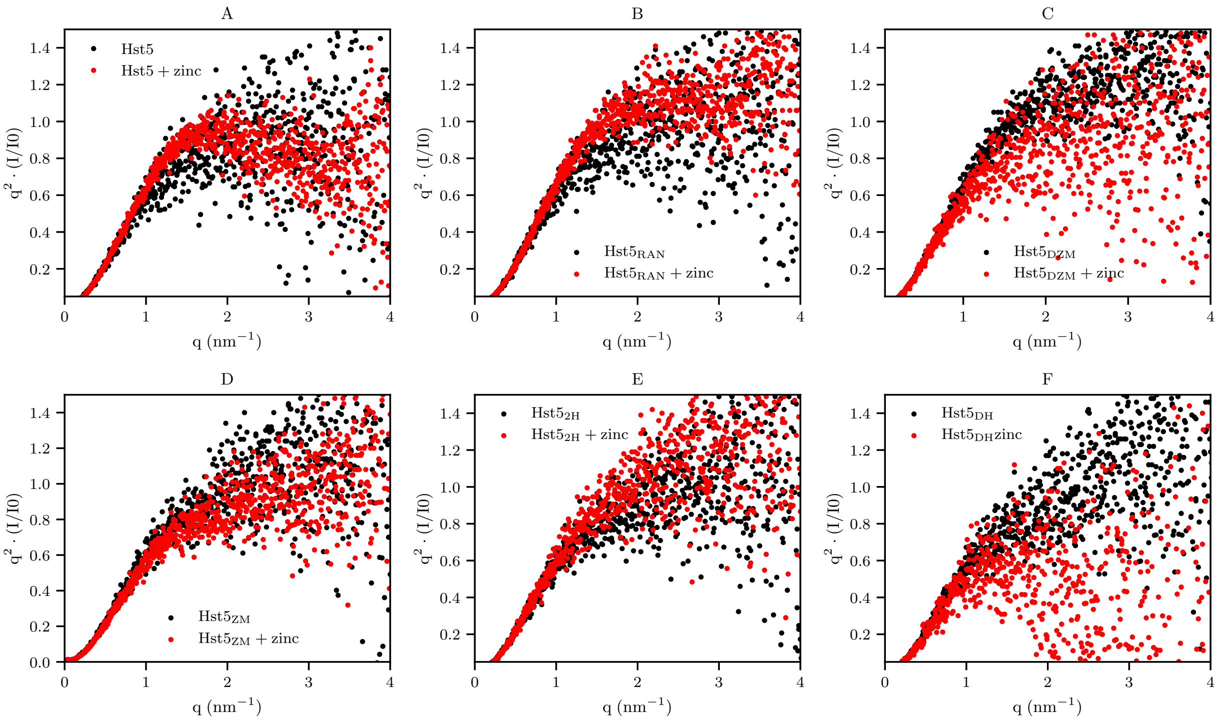

Supplementary Figure 2: **Kratky plots visualising the difference in shape between Hst5 in the absence and presence of zinc. Shown for SAXS data at low concentrations ≈ 1 mg/ml.** Where red dots indicate the absence of zinc and black dots the presence of zinc. A), Hst5 B) Hst5$_{\mathrm{RAN}}$, C)Hst5$_{\mathrm{DZM}}$, D) Hst5$_{\mathrm{ZM}}$, E) Hst5$_{\mathrm{2H}}$, F) Hst5$_{\mathrm{DH}}$.

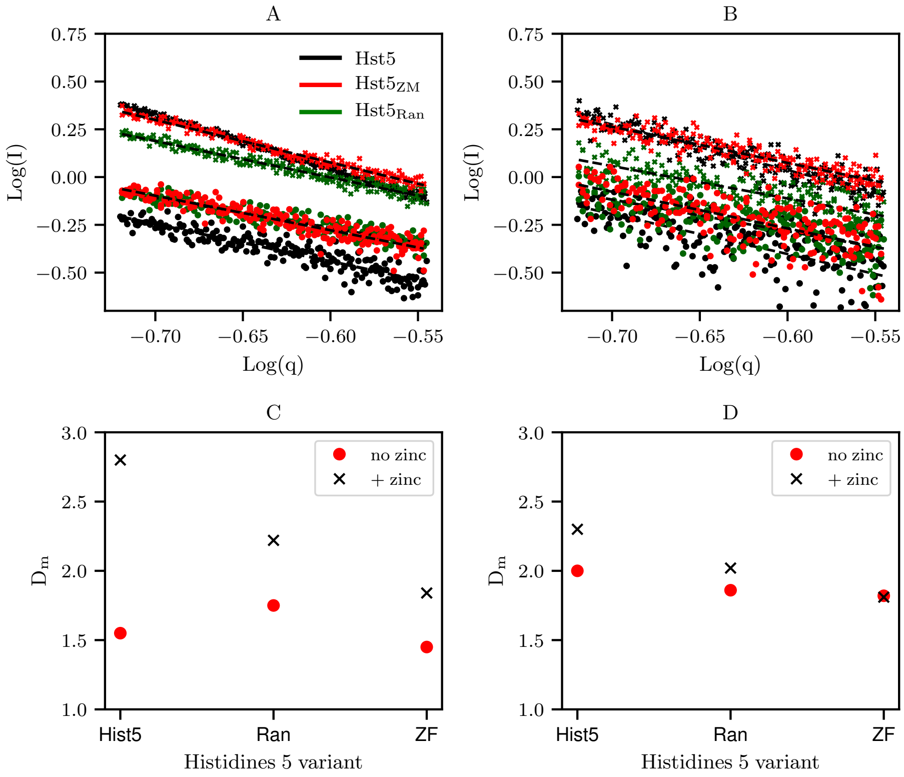

Supplementary Figure 3: **The effect of zinc on the shape of Histatin 5 and selected variants**) SAXS from 5 mg/mL Histatin variants in the absence of zinc (circles) and presence of zinc (squares) plotted as a log-log plot. The dashed lines represent a fit of the powerlaw relationship to the scattering data from which the fractal dimension Dm was estimated. Compared with the low concentration measurements (1 mg/ml) of the same peptides shown in B). C and D) Plot of Dm against the number of hisitidines present in the zinc finger of the different Histatin mutants in the absence of zinc (red circles) and in the presence of zinc (black crosses) corresponding to the concentrations shown in A and B, respectively.

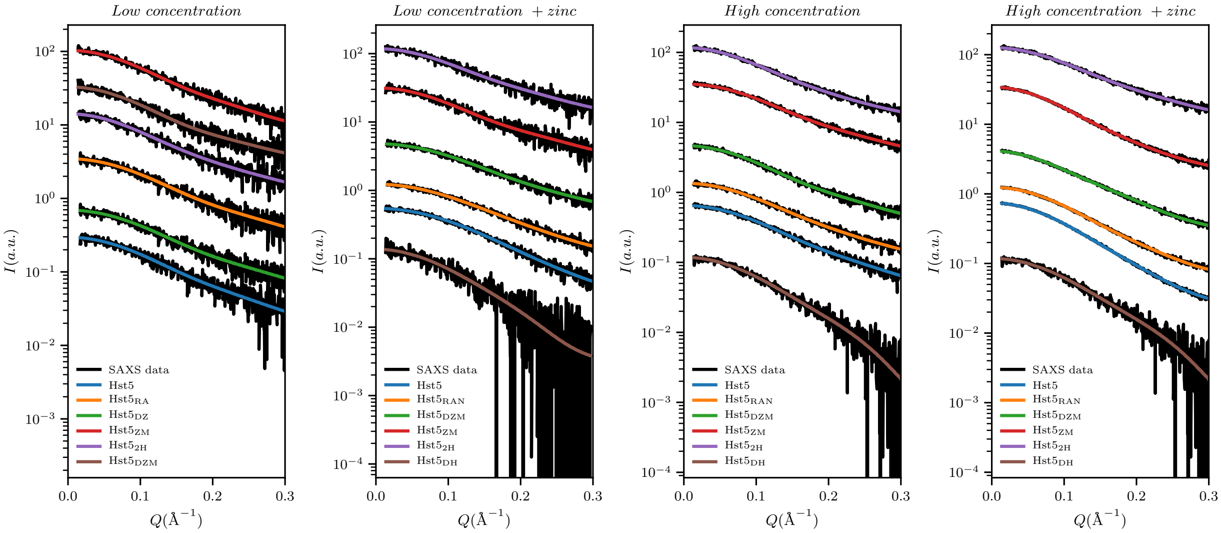

Supplementary Figure 4: **Fits calculated from the DAMMIF scattering envelope shown for each measured sample.** Low concentration corresponds to samples measured at around 1 mg/ml and high concentration to those measured at around 4 mg/ml.

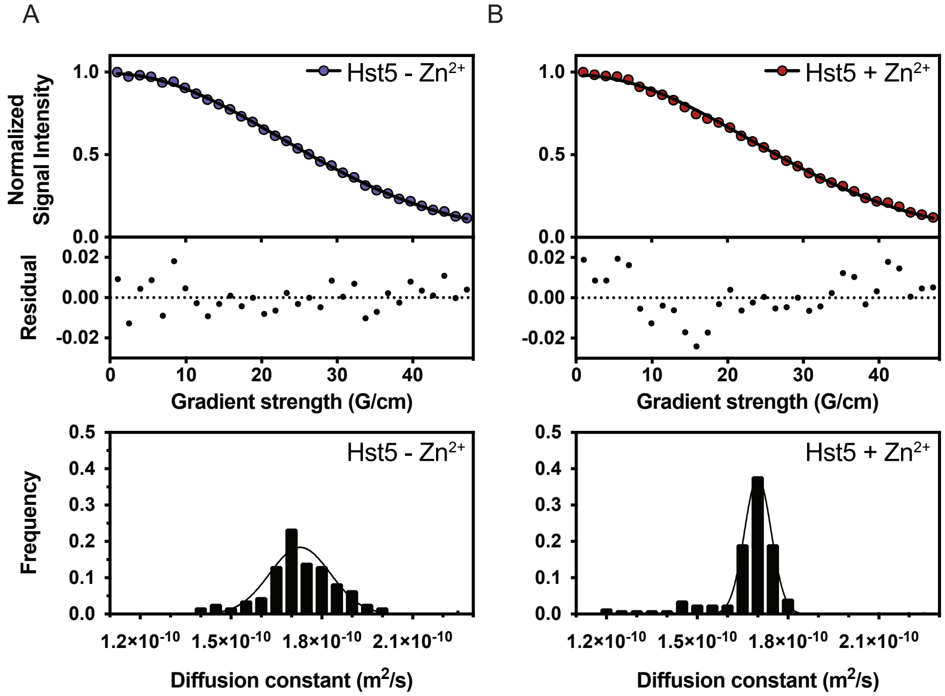

Supplementary Figure 5: **Representative diffusion data measured on Wt Hst5 A) with and B) without Zn^{2+} added.** The upper panels show the signal intensity decay of a single peak with increasing gradient strength and have been fitted to eq. 2. Bottom panels show histograms of the extracted diffusion coefficients from a selection of peaks and have been fitted to a Gaussian function (eq. 3).

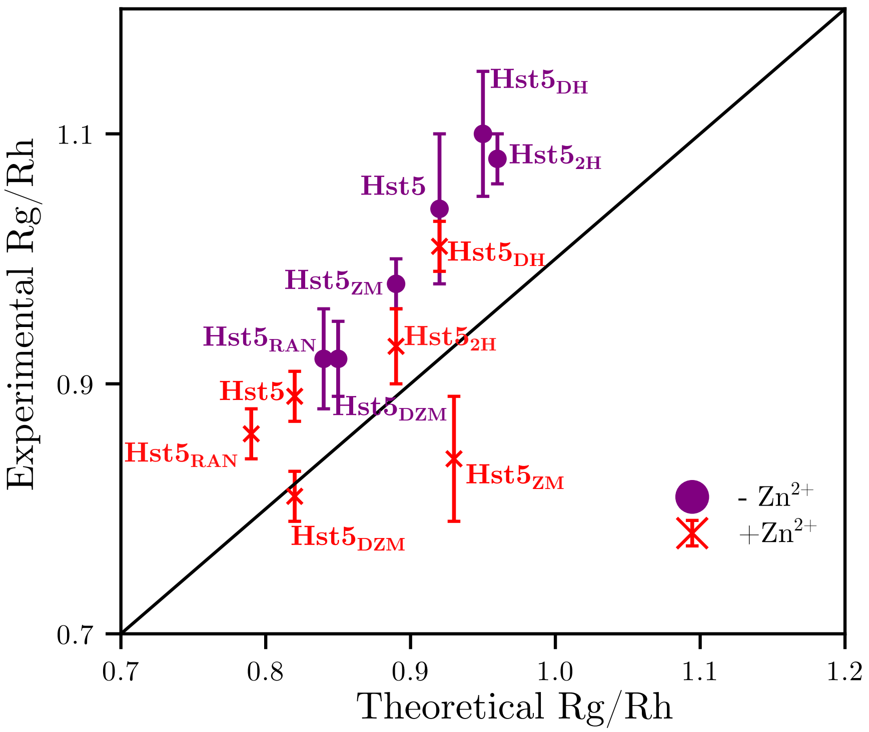

Supplementary Figure 6: **Experimental Rg/Rh ratio compared with theoretical Rg/Rh ratio for the Hst5 variants.** Experimental Rg/Rh ratio determined by SAXS and NMR compared against the theoretical Rg/Rh ratio determined from the empirical relation of Nygaard *et al*[1]. Shown for the Histatin 5 variants in the presence and absence of Zn^{2+}

References

1. Nygaard, M., Kragelund, B. B., Papaleo, E. & Lindorff-Larsen, K. An efficient method for estimating the hydrodynamic radius of disordered protein conformations. *Biophysical journal* **113,** 550–557 (2017).
